# An In Vivo Targeted Deletion of the Calmodulin-Binding Domain from Rice Glutamate Decarboxylase 3 (*Os*GAD3) Increases γ-Aminobutyric Acid Content in Grains

**DOI:** 10.1186/s12284-020-00380-w

**Published:** 2020-03-16

**Authors:** Kazuhito Akama, Nadia Akter, Hinako Endo, Masako Kanesaki, Masaki Endo, Seiichi Toki

**Affiliations:** 1 Department of Life Science, Shimane University, 1060 Nishikawatsu, Matsue, Shimane 690-8504 Japan; 2 Plant Genome Engineering Research Unit, Institute of Agrobiological Sciences, National Agriculture and Food Research Organization, 3-1-3 Kannondai, Tsukuba, Ibaraki 305-8604 Japan; 3 Kihara Institute for Biological Research, Yokohama City University, 641-12 Maioka-cho, Yokohama, 244-0813 Japan

**Keywords:** *Agrobacterium*, Calmodulin-binding domain, CRISPR/Cas9, γ-Aminobutyric acid, Genome editing, Glutamate decarboxylase

## Abstract

**Background:**

Gamma-aminobutyric acid (GABA) is a non-protein amino acid present in all living things. GABA is mainly synthesized from glutamate by glutamate decarboxylase (GAD). In plants the enzymatic activity of GAD is activated by Ca^2+^/calmodulin binding (CaMBD) at the C-terminus in response to various stresses, allowing rapid GABA accumulation in cells. GABA plays a central role in not only stress responses but also many aspects of plant growth and development as a signaling molecules. Furthermore, it is known to be a health-promoting functional substance that exerts improvements in life-style related diseases such as hypertension, diabetes, hyperlipidemia, and so on. Previous reports indicated that CaMBD found plant GADs possess an autoinhibitory function because truncation of GAD resulted in extreme GABA accumulation in plant cells. Therefore, we attempted a genetic modification of rice *GAD* via genome editing technology to increase GABA levels in the edible part of rice.

**Results:**

In this study, we focused on *GAD3*, one of five *GAD* genes present in the rice genome, because *GAD3* is the predominantly expressed in seeds, as reported previously. We confirmed that GAD3 has an authentic Ca^2+^/CaMBD that functions as an autoinhibitory domain. CRISPR/Cas9-mediated genome editing was performed to trim the coding region of CaMBD off from the *OsGAD3* gene, then introducing this transgene into rice scutellum-derived calli using an all-in-one vector harboring guide RNAs and CRISPR/Cas9 via *Agrobacterium* to regenerate rice plants. Out of 24 transformed rice (T_1_), a genome-edited rice line (#8_8) derived from two independent cleavages and ligations in the N-terminal position encoding *Os*GAD3-CaMBD and 40 bp downstream of the termination codon, respectively, displayed a **AK**NQDAAD peptide in the C-terminal region of the putative *Os*GAD3 in place of its intact CaMBD (bold indicates the trace of the N-terminal dipeptides of the authentic CaMBD). A very similar rice line (#8_1) carrying **AK**NRSSRRSGR in *Os*GAD3 was obtained from one base pair deletion in the N-terminal coding region of the CaMBD. Free amino acid analysis of the seeds (T_2_) indicated that the former line contained seven-fold higher levels of GABA than wild-type, whereas the latter line had similar levels to the wild-type, although in vitro enzyme activities of recombinant GAD proteins based on the GAD3 amino acid sequence elucidated from these two lines in the absence of Ca^2+^/bovine CaM were both higher than wild-type counterpart. In addition to high level of GABA in #8_8, the average seed weight per grain and protein content were superior to wild-type and #8_1.

**Conclusions:**

We have successfully established GABA-fortified rice by using CRISPR/Cas9 genome editing technology. Modified rice contained seven-fold higher GABA content and furthermore displayed significantly higher grain weight and protein content than wild-type brown rice. This is the first report of the production of GABA-enriched rice via a genome editing.

## Background

Gamma-aminobutyric acid (GABA) was first discovered in potato tuber tissue (Steward et al. 1949). Today GABA is well known as a major inhibitory neurotransmitter in animals (Roberts and Eidelberg 1960). Although GABA is a non-protein amino acid, it is ubiquitously present both in prokaryotes and eukaryotes (Shelp et al. 1999). Glutamate decarboxylase (GAD) is responsible for the conversion of L-glutamate to GABA, which is the major pathway for the production of GABA in spite of an alternative polyamine pathway associated with GABA synthesis (Shelp et al. 2012).

GABA is unique in plants, because various biotic and abiotic stresses rapidly induce GABA accumulation in cells (Kinnersley and Turano 2000). Baum et al. (1993) first identified the molecular structure of plant GAD from petunia, showing the presence of a Ca^2+^/calmodulin-binding domain (CaMBD) at the C-terminus, indicating Ca^2+^/CaM-dependent activation of petunia GAD, which was subsequently found to be in common in plant GADs. This structure clearly demonstrated that GABA accumulates in plant cells accompanied by elevated Ca^2+^ in response to stresses (Knight et al. 1991). Therefore, plant GAD plays a central role in the regulation of GABA content. Of note, exceptional structures of GADs that are lacking an authentic CaMBD have been reported from rice, apple, and tea (Akama et al. 2001; Trobacher et al. 2013; Mei et al. 2016). A recent study in tea indicated that transcriptional up-regulation of the gene for CaMBD-less GAD in response to anoxic stress is an alternative way to accumulate GABA in cells (Mei et al. 2016).

There have been many studies on putative GABA functions in plants such as a contribution to carbon/nitrogen (C/N) balance, homeostasis of cytosolic pH, defense against insect herbivory, and so on (Bouché and Fromm 2004). Recent studies have implicated GABA as a signaling molecule in plants (Ramesh et al. 2017; Shelp et al. 2017). For example, GABA concentrations increase along the path in the pistil so that pollen tubes correctly reach the ovule (Palanivelu et al. 2003). More recently, a GABA receptor has been identified (Ramesh et al. 2015): the receptor is an aluminum-activated malate transporter, which is activated by anions and negatively regulated by GABA, causing its multiple effects in plants to play a critical role in many aspects of plant growth and development.

On the other hand, GABA is known to be a health-promoting functional substance able to exert improvement of life-style-related diseases such as hypertension, diabetes, hyperlipidemia, and so on. Based on these benefits of GABA, many kinds of GABA-fortified foods, such as water-soaked rice (Saikusa et al. 1994) and anaerobically fermented green tea have been developed (Tsushida et al. 1987). Furthermore, transgenic approaches have been successfully applied to rice and tomato (Shimajiri et al. 2013a; Takayama et al. 2017). Although these plants accumulate sufficient amounts GABA for practical use, strict GMO regulations have hampered their subsequent utilization.

Recently, genome editing technology has shown the potential to induce heritable mutations in a desired genome position, by using various kinds of site-specific nucleases (Gaj et al. 2013). Among these, the clustered regularly interspaced palindromic repeats (CRISPR)/CRISPR-associated protein (Cas) system has rapidly emerged as a powerful and robust genome-editing tool in many organisms including crops (Demirci et al. 2018; Mishra et al. 2018). Two major advantages of genome editing system can be considered: first, accurate and efficient introduction of a mutation can be observed at a targeted site. Second, the modified crops show no difference from those developed through general breeding techniques.

In this study, we have focused on *Os*GAD3, because of its strong expression in rice seeds (Liu et al. 2005). Thus, *Os*GAD3 is suitable as a target gene for modification of the GABA shunt to increase GABA content in an edible part of rice. We mainly evaluated the effect of truncation of the C-terminal Ca^2+^/CaMBD on GABA accumulation in rice grains, by using mutagenesis based on CRISPR/Cas9.

## Results

### Comparison of C-Terminal Regions from Plant GADs

**Fig. 1:**
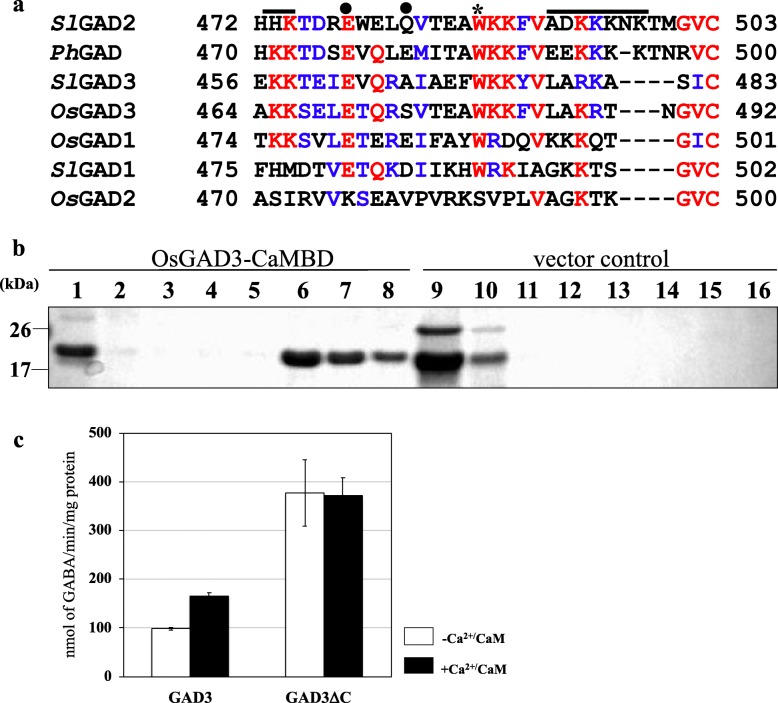
Molecular features of rice GAD3. **a** Alignment of C-terminal regions of putative GADs from plants. Red and blue colors indicate identical amino acids and similar amino acids, respectively**.** Trp (W) and Lys (K) essential for in vitro binding to CaM (Arazi et al. 1995) are indicated by asterisks and a thick line, respectively. The positions of two pseudosubstrate residues (E476 and E480 in *Ph*GAD) (Yap et al. 2003) are indicated by black circles. Os: *Oryza sativa*, Ph: *Petunia hybrida*, Sl: *Solanum lycopersicum*. *Ph*GAD (L16797), *Os*GAD1 (AB056060), *Os*GAD2 (AB056061), *Os*GAD3 (AK071556), *Sl*GAD1 ((AB359913), *Sl*GAD2 (AB359914), *Sl*GAD3 (AB359915). **b** In vitro Ca^2+^/CaM binding ability of recombinant *Os*GAD3. Coding sequence for Ca^2+^/CaMBD of *Os*GAD3 was ligated in-frame with a thioredoxin-coding sequence in an expression vector. Expression of recombinant protein was induced in *E. coli* for purification with an affinity column. The resulting fusion protein (Fr.1) subjected to a CaM-agarose resin separation; effluent fractions (Fr. 2–5) and eluted fractions with EGTA (Fr. 6–8) were all analyzed using 5–20% SDS-polyacrylamide gel electrophoresis to detect protein bands with Coomassie Brilliant Blue staining. As a control, an intact expression vector was used (Fr. 9: vector-encoding protein, Fr. 10–13: effluent fractions, Fr.14–16: eluted fractions). **c** In vitro enzyme assays for recombinant *Os*GAD3 and *Os*GAD3ΔC. The GAD assay was performed to measure GAD production, as described in Akama and Takaiwa (2007). –Ca^2+^/CaM; without Ca^2+^/CaM, +Ca^2+^/CaM; with 0.5 mM Ca^2+^ and 0.1 μM bovine calmodulin (Sigma). Data present the mean ± standard deviation of three independent experiments for both

Plant GADs generally possesses a Ca^2+^/CaM-binding domain at the C-terminus, which plays a critical role in the regulation of GAD enzymatic activity (Baum et al. 1993), although several exceptions, including *Os*GAD2 (Fig. 1a), have been reported so far (Akama and Takaiwa 2007). As shown in Fig. 1a, the overall similarity of the C-terminal region in plant GADs is quite low, but several conserved motifs are present: the highly conserved tryptophan (W) residue is located at the center of the domain and 2–4 lysine (K) repeats including arginine (R) are observed at independent locations at the C-terminus of GAD. In *Petunia* GAD, the most characterized enzyme of the family, E476 and E480 are thought to work as pseudosubstrates of glutamate (Yap et al. 2003). The corresponding amino acid to E476 is highly conserved in other plant GADs, implying an identical function. Because both *Ph*GAD and *Os*GAD1 have been reported to have an in vitro Ca^2+^/CaM binding ability (Baum et al. 1993; Akama et al. 2001), it was speculated that *Os*GAD3 potentially possesses the same ability.

### Ca^2+^/CaM-Binding Domain of *Os*GAD3 Prerequisite for In Vitro CaM Binding

In order to confirm the Ca^2+^/CaM-binding ability of the C-terminal domain of *Os*GAD3, we constructed an expression vector carrying a DNA fragment for the C-terminal domain of *Os*GAD3, followed by immobilization into *E. coli* strain BL21 (DE3) pLysS. Recombinant protein carrying a polyhistidine-tag was induced for purification using a nickel-affinity resin. The purified protein was incubated with bovine CaM agarose beads in the presence of Ca^2+^, then the beads were washed with an excess amount of Ca^2+^-containing buffer. Finally, the bound protein was eluted with EGTA-containing buffer. As shown in Fig. 1b, the recombinant protein with the C-terminal region of *Os*GAD3 was not detected in the washing fractions (Fr. 3 to 5) but was detected in the elution fractions (Fr. 6 to 8). The opposite was the case with the vector control. An in vitro experiment showed that *Os*GAD3 has an ability to bind to Ca^2+^/CaM, suggesting that *Os*GAD3 is in fact a Ca^2+^/CaM-dependent enzyme like other typical GADs in plants.

### In Vitro GAD Assay of Recombinant *Os*GAD3 and its Truncated Version

In order to explore the effect of the C-terminal Ca^2+^/CaMBD on *Os*GAD3 enzyme activity, we produced two different recombinant proteins, wild-type *Os*GAD3 and C-terminal truncated *Os*GAD3ΔC that lacked the Ca^2+^/CaMBD. As shown in Fig. 1c, a wild-type enzyme was activated about 1.5 times in the presence of Ca^2+^/bovine CaM at pH 7.0. In contrast, *Os*GAD3ΔC showed much higher activity, irrespective of Ca^2+^/CaM. This indicated that the C-terminal domain of *Os*GAD3 plays a role as a strong autoinhibitory domain, and thus truncation of this domain causes the enzyme to act constitutively, with higher activity at least in vitro.

### Strategy for In Vivo Truncation of *Os*GAD3-CaMBD Via Genome Editing

**Fig. 2:**
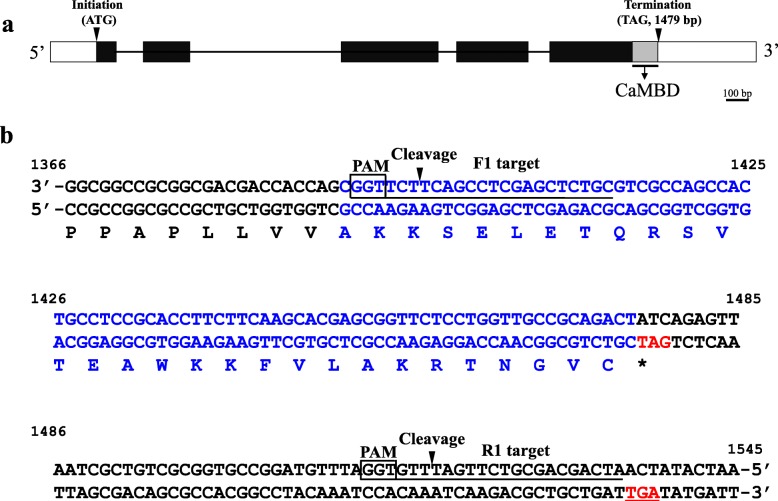
Schematic diagram of *OsGAD3* (Os03g0236200) gene structure and gRNA target sit for CRISPR/Cas9-mediated mutagenesis of *OsGAD3*. **a** Exons, introns, and UTRs are indicated by solid boxes, lines and blank boxes, respectively. Upstream and downstream arrows indicate translation initiation and termination sites, respectively. The numbers in brackets indicate the distance of an exon sequence to the initiation codon (ATG). A gray box indicates a calmodulin-binding domain (CaMBD). Scale corresponds to 100 bp. **b** Downstream region of *OsGAD3* gene from nucleotide position 1366 to 1545. The amino acid sequence is shown below nucleotide sequence. An asterisk indicates a termination codon. Nucleotide sequence and its translated amino acid corresponded to a CaMBD is indicated in blue. Two target sequences (F1 target and R1 target) of the CRISPR/Cas9 are underlined. Boxes show the protospacer adjacent motif (PAM: 5′-NGG-3′) sequences. Black arrows show a putative cleavage sites of gRNA. Two termination codons are indicated in red. The former is an authentic termination codon and the latter one with a line is used after precious cleavage at the two cleavage sites and joining

Figure 2a shows the exon/intron structure of *OsGAD3* gene, where a putative CaMBD is located in the proximal region of the last exon. In order to remove the coding region for *Os*GAD3-CaMBD, guide RNAs (gRNAs) were designed for trimming off the CaMBD-coding region, as shown in Fig. 2b. The reason to take a mild strategy for its trimming but not introduce a premature termination codon via a frameshift mutation is that a trace of the coding region of *Os*GAD3-CaMBD between a premature termination codon and a transcriptional terminator of *Os*GAD3 and/or premature termination codon itself may have a possibility to influence the mutated *GAD3* gene at the transcription and/or translation level. It is expected that cleavage of F1 and R1 will induce a 122 bp deletion, thus terminating the translation at the underlined termination codon (Fig. 2b). An adequate DNA repair resulted in the removal of almost an intact CaMBD and adding 6 amino acid residues.

### Plant Transformation Via *Agrobacterium* and Screening

**Fig. 3:**
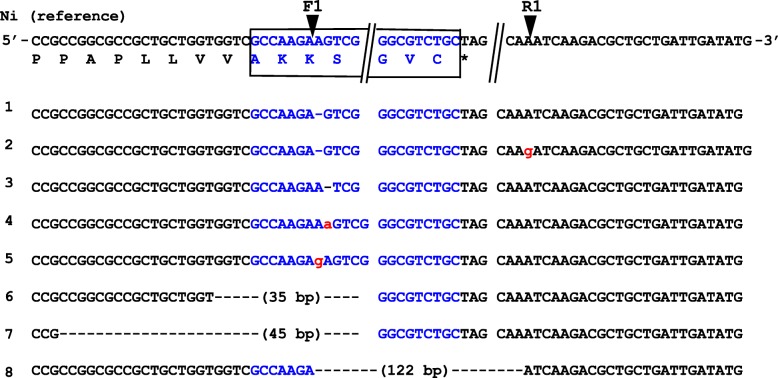
DNA sequence of target regions in *Os*GAD3. Ni (wild-type variety Nipponbare) indicates nucleotide and amino acid sequences of the *OsGAD3* gene in the target region of CRISPR/Cas9. Two black arrows (F1 and R1) indicate putative cleavage sites of CRISPR/Cas9. The CaMBD is indicated in blue. Double slash marks indicate the presence of sequence gaps. No. 1 to 8 indicate examples of mutations observed in the coding region of OsGAD3-CaMBD. Small letters in red indicate an insertion. Hyphens indicate a deletion. Base pairs (bp) in brackets indicate the length of the deletion

A Ti plasmid vector harboring two kinds of gRNAs and a Cas9 gene cassette was introduced into and *Agrobacterium* strain to infect calli derived from rice scutellum. From introduction into *Agrobacterium* strains (i.e., vector #8: F1 and R1 on gRNA combination), finally yielding 24 lines (T_1_). After harvesting, T_1_ seeds (six pooled seeds) were analyzed on GABA content and targeted DNA editing (Table S1). For GABA content, most lines showed similar levels to wild-type Nipponbare (Ni), whereas several lines had increased about five-fold, compared with Ni. DNA analysis of targeted regions from T_1_ plants revealed 8 different mutagenesis patterns (Fig. 3). In the T_1_ generation, most of lines showed bi-allelic or chimeric genotypes (Table S1).

### Analysis of Genome-Edited Rice T_2_ Lines on DNA Sequences and GABA Content

In sequence analysis of the T_2_ generation, pattern 1 and pattern 8 were mainly extracted from #8_1 and #8_8, respectively. We established a homozygous line for each of these to use for further analyses. As shown in Table 1, patterns 1 and 8 were very similar, i.e., common N-terminal AK and artificial peptides of 9 and 6 amino acids, respectively. The former was derived from a one base pair deletion in the F1 target site, whereas the latter was derived from the expected deletion between F1 and R1 (Fig. 3). Of note, a free amino acid analysis of these T_2_ seeds about GABA demonstrated that #8_1 was almost the same level as wild-type (Ni) while #8_8 was seven-fold higher than Ni (Table 2). Surprisingly, several amino acids such as Asn, Asp and Glu significantly increased in #8_1, compared with wild-type Ni. We extracted free amino acids from leaf, stem, and root tissues of rice seedling to compare the GABA content. Table 3 shows that GABA contents in stems were similar among the three lines, whereas leaves in line #8_8 and roots in lines #8_1 and #8_8 contained about half of wild-type and three- to five-fold GABA more than wild-type, respectively.

**Table 1:** Amino acid sequence of target regions

Class	Amino acid sequence
Ni	PPAPLLVV**AKKSELETQRSVTEAWKKFVLAKRTNGVC**
1	PPAPLLVV**AK***SRSSRRSGR*
2	PPAPLLVV**AK***SRSSRRSGR*
3	PPAPLLVV**AK***NRSSRRSGR*
4	PPAPLLVV**AKK***VGARDAAVGDGGVEEVRARQEDQRRLLVSISDSATAYKSTNQDAAD*
5	PPAPLLVV**AK***RVGARDAAVGDGGVEEVRARQEDQRRLLVSISDSATAYKSTNQDAAD*
6	PPAPLLV*GDGGVEEVRARQEDQRRLLVSISDSATAYKSTNQDAAD*
7	P**QRSVTEAWKKFVLAKRTNGVC**
8	PPAPLLVV**AK***NQDAAD*

**Table 2:** Free amino acid content in rice grains

Amino acid	Ni	#8_1		#8_8	
			(nmol/g FW)		
Ala	431.2 ± 176.4	503.0 ± 94.2	**1.2**	905.4 ± 322.4**	**2.1**
Gly	159.9 ± 55.5	131.7 ± 59.6	**0.8**	106.9 ± 19.2	**0.7**
Val	66.4 ± 18.2	81.0 ± 30.1	**1.2**	146.3 ± 44.8**	**2.2**
Leu	17.7 ± 6.2	53.0 ± 14.2**	**3.0**	54.9 ± 21.2**	**3.1**
Ile	10.2 ± 4.1	6.0 ± 3.5	**0.6**	53.1 ± 19.8**	**5.2**
Ser	281.0 ± 72.7	25.0 ± 17.8**	**0.1**	448.3 ± 94.2**	**1.6**
Pro	210.6 ± 65.1	127.7 ± 47.9	**0.6**	139.8 ± 59.6	**0.7**
Asn	1758.6 ± 412.2	5018.0 ± 2805.0**	**2.9**	566.5 ± 302.1**	**0.3**
Asp	1042.7 ± 245.6	3480.3 ± 1674.0**	**3.3**	2289.0 ± 813.9**	**2.2**
Met	549.7 ± 48.1	146.3 ± 57.0*	**0.3**	1524.5 ± 371.2**	**2.8**
Glu	1866.6 ± 588.6	3685.0 ± 876.5**	**2.0**	2898.9 ± 1105.2*	**1.6**
Phe	19.7 ± 7.2	24.3 ± 6.0	**1.2**	55.0 ± 18.8**	**2.8**
Gln	19.2 ± 8.9	130.7 ± 58.4**	**6.8**	ND	
His	34.9 ± 23.3	253.0 ± 133.9**	**7.2**	ND	
Tyr	24.3 ± 10.5	36.3 ± 17.0	**1.5**	25.2 ± 11.1	**1.0**
Trp	42.9 ± 12.6	4.0 ± 3.4**	**0.1**	13.6 ± 4.8**	**0.3**
GABA	32.0 ± 10.1	18.3 ± 10.2	**0.6**	223.5 ± 93.9**	**7.0**

Value:average ± sandard deviation

Bold:fold against Ni

*ND* not detected

* *P* < 0.05 and ** *P* < 0.01 versus Ni control

**Table 3:** GABA content in rice plants

Tissue	Ni	#8_1	#8_8
	(nmol/g FW)		
Leaf	202.1 ± 1.1	165.5 ± 18	124.6 ± 25.4*
Stem	38.2 ± 9.1	89.8 ± 44.9	46.9 ± 14.4
Root	69.0 ± 12.0	250.2 ± 91.4*	402.7 ± 26.4*

Value:mean ± standard deviation

*P < 0.05 versus Ni control

### mRNA Analysis of *GAD3* in Wild-Type, #8_1, and #8_8 Rice Plants

**Fig. 4:**
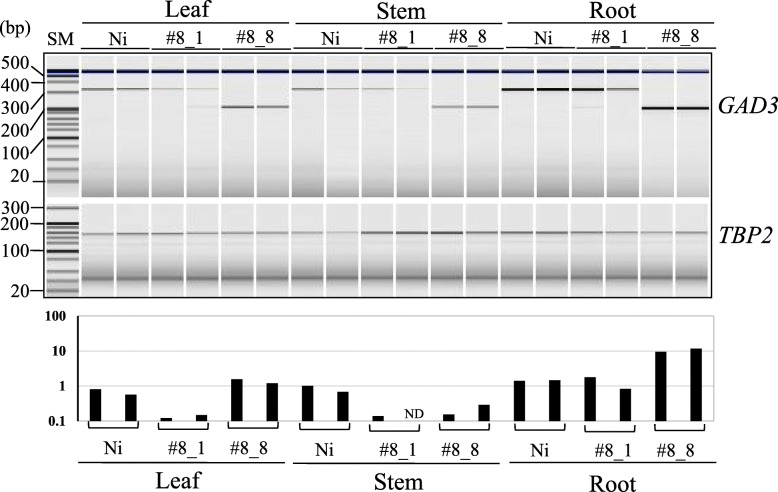
Semi-quantitative RT-PCR analysis of RNA transcribed from *OsGAD3* genes in wild-type (Ni) and genome-edited rice (lines #8_1 and #8_8 in Table S1). Total RNA was isolated from the tissues indicated above the lanes. Single-stranded cDNA synthesized from each RNA (2 μg) with an oligo-dT primer using reverse transcriptase was amplified with an appropriate primer set (Table S2). PCR conditions were as follows: cycles of 95 °C 30 s, 60 °C 30 s, 72 °C 30 s were repeated by 27 times for *GAD3* and 25 times for *TBP2*. *OsGAD3* and *TBP2* indicate reactions with primers GAD3-F57 and GAD3-R379 or TBP2-F and TBP2-R as an internal control, respectively. Samples were analyzed using a DNA-500 of MULTINA (Shimadzu, Kyoto, Japan). Graph shown at the bottom indicates the ratio of band intensity of *GAD3* to that of *TBP2*. SM shows a DNA size maker. ND: not detected

It was speculated that the expression level of the *GAD3* in these plants is critical for the GABA content in each tissue. The RiceExpro database (Sato et al. 2013) shows that *GAD3* expression level in the roots is significantly higher than in leaves and stems in vegetative tissues. In order to determine the correlation between GABA content and *GAD3* mRNA expression, semi-quantitative reverse transcriptase polymerase chain reaction (RT-PCR) analysis of mRNA extracted from the three different tissues was compared. A *TATA-binding protein 2* (*TBP2*) was used as an internal control because *TBP2* was relevant to a *glyeraldehyde-3-phosphate dehydrogenase* (*GAPDH*) commonly used as a reference gene in plants (data not shown). Figure 4 shows that expression level in roots was higher than that in leaves and stems, which was almost consistent with the GABA content in the three tissues (Table 3). Moreover, it was clear that the expression in leaves and stems from #8_1 was lower than those from Ni and #8_8 (Fig. 4).

### In Vitro Assay of GAD Enzymes from Genome-Edited #8_1 and #8_8 Plants

**Fig. 5:**
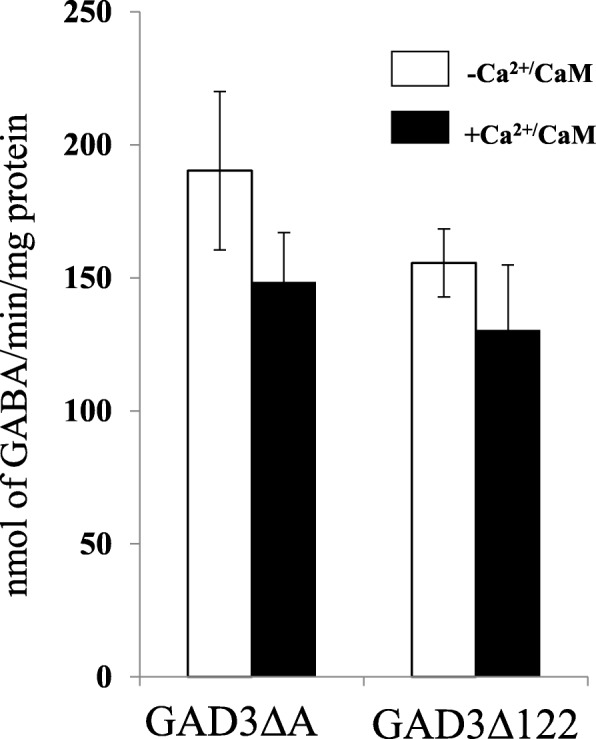
In vitro enzyme assay for recombinant *Os*GADs based on the nucleotide sequence from genome-edited rice plants (#8_1 and #8_8). The GAD assay was performed to measure GAD production as in Fig. 1c. –Ca^2+/^CaM; without Ca^2+^/CaM, +Ca^2+^/CaM: with 0.5 mM Ca^2+^ and 0.1 μM bovine calmodulin (Sigma). Data present the mean ± standard deviation of three independent experiments for both

Because we could not detect any difference in GAD specific activity among seeds or roots of the three rice lines (data not shown), we prepared recombinant GAD3 proteins from #8_1 and #8_8, carrying the C-terminal portion of #8_1: **AK**SRSSRRSGR (GAD3ΔA) and #8_8 **AK**NQDAAD (GAD3Δ122) using an *E. coli* expression system. Of note, these two recombinant proteins were expressed in large amounts, mostly in the soluble fraction, in contrast to wild-type *Os*GAD3 and *Os*GAD3ΔC, in which the soluble fraction was recovered in small amounts. As shown in Fig. 5, both proteins were Ca^2+^/CaM-independent like *Os*GAD3ΔC, but, they displayed lower activity than *Os*GAD3ΔC (Fig. 1c). Comparing these two genome edited versions, a truncated version of *Os*GAD3ΔA from #8_1 showed slightly higher activity than that from #8_8.

### Pleiotropic Effect of Genome-Edited *GAD3* on the Characteristics of Seeds

We analyzed the GABA content in seeds and vegetative tissues among wild-type, #8_1, and #8_8 and showed significant accumulation in the grains and roots of genome-edited lines. Fig. S1 summarizes several other features in seeds, notably, #8_8 had significantly higher in seed weight per grain (12%) and protein contents (20%) than Ni or #8_1 and Ni, respectively.

## Discussion

In this study we established rice lines where the coding region of CaMBD was deleted from *OsGAD3* gene via a genome-editing procedure. Rice contains five *GAD* genes, among which *GAD3* is known for its strong expression in seeds (Liu et al. 2005), implying that *GAD3* is a possible target for metabolic engineering of the GABA shunt. Furthermore, Fig. 1b shows that *Os*GAD3 has an authentic CaMBD, as found in most plant GADs, meaning that it is activated in a Ca^2+^/CaM-dependent manner. In fact, an in vitro enzyme assay of recombinant *Os*GAD3 indicated Ca^2+^/CaM-dependent activation, whereas a removal of CaMBD resulted in an approximately three and half-fold increase in GAD activity at pH 7.0, irrespective of Ca^2+^/CaM (Fig. 1c). This indicated that the C-terminal domain functions as a strong autoinhibitory domain. There are several reports on the enzyme activity of CaMBD-truncated plant GAD (GADΔC) in vitro. For example, the enzyme activity of recombinant tea GAD1ΔC showed an approximately 1.8-fold increase in GABA compared with wild-type GAD1 at pH 5.8 (Mei et al. 2016), and tomato GADsΔC enhanced their activities from 10- to 16-fold at pH 7.0 (Nonaka et al. 2017). *Os*GAD3 is the similar responsiveness to CaMBD-truncation as well.

Therefore, it is expected that an in vivo truncation of CaMBD causes rice plants to increase GABA content as reported (Baum et al. 1996; Takayama et al. 2017). For in vivo mutagenesis, we attempted an intact deletion of the coding region for Ca^2+^/CaMBD using CRISPR/Cas9, but not by introducing a premature termination codon before the coding region. We established 22lines (Table S1). Out of 17 lines analyzed at the DNA level, seven lines contained the expected 122 bp deletion (41%). The remaining lines were mostly single-base deletions and insertions (Fig. 3). Wang et al. (2017) reported the deletion of a target gene in rice via CRISPR/Cas9, and the deletion frequency correlated with the target size. For example, over 21% for a 430 bp target deletion but only 9% for a 10 kbp target. It is clear that shorter deletions were achieved with high frequency. In addition, almost T_1_ lines contained two or three mutations including a wild-type, showing not only bi-allelic but also chimeric forms. Mikami et al. (2015) reported bi-allelic mutations frequently using an all-in-one expression vector carrying Cas9/gRNA. Because of the relatively longer selection period for calli from rice, plant lines with chimeric genotype may tend to regenerate as this study.

As expected, the GABA content in #8_8 was seven-fold higher than wild-type Ni (Table 2). Of note, a very similar mutant #8_1, which was caused by a one base pair deletion causing a truncation of CaMBD, was similar to the wild-type GABA content (Table 2). There was a subtle difference between these in the resulting peptide (Table 1). In vitro GAD activity of recombinant proteins #8_1 and #8_8 were both higher than GAD3 in the absence of Ca^2+^/CaM, meaning that the putative proteins created from genome-editing have potentially higher GAD activity in vivo than the wild-type (Figs. 1c and 5). However, the GABA contents in vegetative tissues produce different results (Table 3), and there was no clear tendency among leaves and stems among wild-type Ni and genome-edited lines, but the roots, #8_1 and #8_8 accumulated much higher GABA levels than Ni. Semi-quantitative RT-PCR indicated that the relative expression level in roots was high in *GAD3* in each line. These results suggested that lower levels of transcription of *GAD3* may cancel the effect of truncation of CaMBD in leaves and stems, due to relatively higher expression levels of the other *GAD* genes (see Fig. S3). On the other hand, because of higher expression in the roots, the truncation effect can be effectively expressed. Nevertheless, comparison of mRNA expression in two genome edited lines demonstrated that #8_1 displayed relatively lower expression than another line #8_8 in all tissues (Fig. 4). Therefore, a decrease in *GAD3* transcription and/or instability of its RNA in #8_1 may be the main reason for the lower GABA level than in #8_8 even in seeds. Supplementary data shown in Fig. S2 seems to support this notion (i.e., *GAD3* mRNA level in seeds: #8_8 > Ni > #8_1). Furthermore, the expression profiles of remaining four rice *GAD* genes indicate that only *GAD1* was strongly expressed in seeds, in which expression levels in the genome-edited lines were almost the same each other (Fig. S3). This strongly suggests that *GAD3* expression level is a critical to determine GABA content at least in the genome edited seeds, because enzyme activities of these were not significantly different (Fig. 5). Hori and Watanabe (2007) reported that the length of the 3′-untranslated region (UTR) plays a critical role in mRNA stability in plants. The appearance of a premature amber codon caused a relatively long 3′-UTR in #8_1, which may have affected *GAD3* mRNA stability. Although we have no explanation for the up-regulation of mutated *GAD3* in #8_8, Mei et al. (2016) reported that not only activation of GAD activity but also up-regulation of *GAD* transcription in response to multiple stresses is critical for the accumulation of GABA in tea plants.

As described above, genome-edited GAD3 proteins from #8_1 and #8_8 showed relatively high activity (Fig. 5). Zik et al. (2006) reported that in *petunia* GAD-CaMBD, deletion of the N-terminal 18 amino acids decreased activity by one-quarter compared with wild-type but with sustained Ca^2+^/CaM-dependency. Conversely, deletion of the C-terminal 9 amino acid decreased activity by 1/20 without a Ca^2+^/CaM response. OsGAD3ΔA (#8_1) and OsGAD3Δ122 (#8_8) both contain the same proximal dipeptide (AK) of CaMBD, but remaining the 6 or 9 peptides were completely different (Table 1). Extension of the sizes of these peptide may not interfere with enzyme activity. Of note, our ongoing experiment on *OsGAD1* by genome edition demonstrated that the presence of the N-terminal five peptides of CaMBD with three tail amino acids drastically decreased its in vitro enzyme activity (in preparation). Taken together, subtle differences in several amino acids remaining in the C-terminal region of *Os*GADs may still play a critical role in the regulation of enzyme activity itself.

Nonaka et al. (2017) reported on targeted mutagenesis in tomato fruits: they modified two *GAD* genes by removing the coding region of CaMBD using the CRSIPR/Cas9 system. Genome-edited tomato accumulated 7- to 15-fold more GABA in fruits than wild-type. In spite of non-desirable effects such as dwarf tendency or smaller plant and fruit size and yield, the resulting products were effective for controlling hypertension via daily intake. Genome-edited rice developed in this study was seven-fold higher levels of GABA than wild-type brown rice. Although milled rice was not examined for GABA content yet, a significant reduction of it might not be avoided due to the higher promoter activity of *OsGAD3* in embryos (Sato et al. 2013).

In parallel to the GABA analysis, not only the average weight of brown rice from #8_8 but also the protein composition was significantly higher than Ni and #8_1 (Fig. S1). Of note, *OsGAD3* promoter::β-glucuronidase (*GUS)* reporter analysis demonstrated that *OsGAD3* was predominantly expressed in the vascular bundle from lateral roots, leaves, and embryo and scutella (Fig. S4). Interestingly, levels of Asp, Asn and Glu significantly increased in #8_1 grains, while they all decreased in #8_8 grains (Table 2). These amino acids play an critical role not only in protein synthesis but also starch synthesis via GABA shunt and TCA cycle (Fait et al., 2011). Therefore, relatively lower level of amino acids observed in #8_8 may partially explain increment in protein content and seed weight. The expression patterns observed in *OsGAD3* indicated that GABA may be synthesized in vascular bundles to translocate via them or Glu translocated via vascular bundles is converted to GABA near them, where *Os*GAD3 has a prominent role in GABA production. Most probably tissue-specific expression patterns of *OsGAD3* shown in Fig. S4 and activated GAD3 enzyme might be both important to exert their effects on seed quality to improve as well.

On the other hand, it has been reported that overexpression of *FLO2* (*FLOURY ENDOSERPM2*) enlarged the size of grains significantly, increasing grain weight and storage proteins (She et al., 2010). It has been shown that high-temperature stress during the seed maturation reduced the accumulation of storage starch in rice grains (Yamakawa et al., 2007). *FLO2* expression was up-regulated by high-temperature stress, supporting idea that *FLO2* plays an important role in the trait for tolerance to high-temperature stress (She et al., 2019). Interestingly, among five *GAD* gene family in rice, only *GAD3* gene was induced by high-temperature stress (Akama, unpublished data). This common feature between *FLO2* and *GAD3* may be a clue to investigate a novel function of *GAD3*. Further analysis of *OsGAD3* will be required for dissection of the relationship between increase in grain productivity and *OsGAD3* function as well as improvement in GABA content.

## Conclusions

Here, we have demonstrated that C-terminal truncation of the coding region of *OsGAD3* was successfully done using CRISPR/Cas9. We developed a new rice line containing seven-fold higher content of GABA than wild-type and displayed improvements in the quality of rice grain such fresh weight per grain and protein concentration.

## Methods

### Plant Materials and Growth Conditions

*Oryza sativa* L. cv. Nipponbare (Ni) was used in this study. Cultivation conditions for rice plants comprised a growth chamber and a naturally lighted non-containment greenhouse (40 m^2^ in area) located in Matsue-city, Japan, as described in Akama et al. (2009).

### Plasmid Construction

In this study, three different kinds of expression vectors were constructed, as follows:

i) *Os*GAD3-CaMBD for an in vitro Ca^2+^/CaM binding assay: The coding region for *Os*GAD3-CaMBD (**AKKSELETQRSVTEAWKKFVLAKRTNGVC)** was amplified by PCR using primer set CaMBD-F and CaMBD-R (Table S2) from *Os*GAD3 cDNA (AK071556) as a template. The resulting fragment was subcloned into pET32a (Novagen) at the *Nco*I and *Eco*RI sites.
ii) Genome-editing vectors for truncation of the C-terminal region of *Os*GAD3: Target guide RNA (gRNA) sequences were designed using the CRISPR-P program (Lei et al. 2014) in the coding region for *Os*GAD3-CaMBD (for gRNA-F1) and downstream (for gRNA-R1) (Fig. 2b). One pair of the synthesized 20-nucleotide target sequences for gRNA-F1 and gRNA-R1 were annealed to make double-stranded DNA, respectively, inserted into the *Bbs*I site of the gRNA cloning vector pU6gRNA (Mikami et al. 2015). A *Pvu*II and *Asc*I fragment from pU6gRNA carrying gRNA-R1 was inserted into pU6gRNA carrying gRNA-F1 via the *Eco*RV and *Asc*I sites, resulting in pU6gRNA_F1 and R1. A *Pvu*II and *Asc*I fragment from this pU6gRNA derivative was inserted at the *Asc*I and *Plm*I sites of a Ti plasmid pZDgRNA_Casver.2_HPT (Mikami et al. 2015) for rice transformation.
iii) *E. coli* expression vector for *Os*GAD3 protein and its derivative proteins for enzyme assays: Four different kinds of protein expression vectors were constructed as follows: A full-length *Os*GAD3 cDNA was used as a PCR-amplification template for the production of two different sizes of coding region of *Os*GAD3. One was a full-length cDNA and the other was a 3′-truncated one. Each PCR product obtained with appropriate primer sets (Table S2) was cloned into the *Nco*I and *Eco*RI sites of pET32a vector, yielding pET32a::*Os*GAD3 and pET32a::*Os*GAD3ΔC. The remaining two plasmids contained additional coding sequences for **AK**SRSSRRSGR (AK9aa) and **AK**NQDAAD (AK6aa) at the C-terminal end of *Os*GAD3ΔC, respectively. Bold indicates the N-terminal dipolypeptides in *Os*GAD3-CaMBD. They were amplified from template DNA of *OsGAD3* cDNA #8_1 and #8_8, as described below, with the appropriate primer set shown in Table S2. These two cDNA fragments from lines #8_1 and #8_8 were inserted in the *Nco*I and *Eco*RI sites of a pET32a vector, designated as pET32a::*Os*GAD3ΔA and pET32a::*Os*GAD3Δ122, respectively.

### Bacterial Culture, Recombinant Protein Induction of pET32a-Derived Expression Vectors and Protein Purification

After sequence confirmation, pET32a-derived expression vectors carrying a coding region for *Os*GAD3-CaMBD, full-length *Os*GAD3, *Os*GAD3ΔC, *Os*GAD3ΔA, and *Os*GAD3Δ22 cDNAs, respectively, were each introduced into *E. coli* strain BL21 (DE3) pLysS (Novagen). Bacterial culture, protein induction and recombinant protein purification were carried out essentially as in Shimajiri et al. (2013b).

### *Agrobacterium*-Mediated Rice Transformation

Each of the binary vectors was introduced into *Agrobacterium* strain EHA105 (Hood et al. 1993). Transformation of rice calli via *Agrobacterium*, selection of calli with 50 mg/L of hygromycin B, and subsequent regeneration were done essentially as described in Hiei et al. (1994).

### Characterization of Genome-Edited Rice Plant Lines

Six rice grains pooled from each transgenic line (T_1_ generation) were ground to a fine powder with MicroSmash (Tomy, Tokyo, Japan). Then, 20 mg of powder was subjected to the isolation of free amino acids with 8% (v/v) of trichloroacetic acid, as described in Akama et al. (2009). In parallel, an aliquot of rice powder was used for the isolation of total DNA in accordance with Doyle and Doyle (1990). The target sequence regions of *OsGAD3* in genome-edited rice plants were PCR-amplified with appropriate primer sets (Table S2), then subcloned in pBluescript II KS (+) (Stratagene) for sequence determination.

### In Vitro Ca^2+^/Calmodulin Binding Assay

Recombinant proteins harboring *Os*GAD3-CaMBD were used for in vitro Ca^2+^/calmodulin binding assays, as described in Akama et al. (2001).

### In Vitro GAD Enzyme Assay

An in vitro GAD enzyme assay (100 mM *bis-tris*-HCl at pH 7.0 as a reaction buffer) for soluble rice GAD protein series extracted from *E. coli* and purified with a histidine-tag purification resin, was performed in accordance with Akama and Takaiwa (2007). Determination of GABA production from its substrate glutamate used GABase (Sigma) based on Akama et al. (2009).

### Amino Acid Analysis by Gas Chromatography/Mass Spectrometry

GABA content in T_1_ generation rice grains and free protein amino acids including GABA in T_2_ generation plants were determined in accordance with Kowaka et al. (2015).

### RNA Extraction and RT-PCR

Total RNA was isolated from tissues (leaf, stem and root) of 2-week-old rice seedlings (wild-type Ni, #8_1 and #8_8) using Sepasol RNAI Super G (Nacalai Tesque, Japan). Single-stranded cDNAs coding for GAD3 and its truncations were synthesized from total RNA as the template using reverse transcriptase (ReverTra Ace, TOYOBO, Osaka, Japan). cDNAs were PCR-amplified with primer set GAD3-F57 and GAD3-R379 (Table S2). As an internal control, *TATA-binding protein 2* (*TBP2*) was used as a target to amplify with primers TBP2-F and TBP2-R (Table S2). The PCR products were analyzed using an automated gel electrophoresis system (MULTINA, Shimadzu, Kyoto, Japan).

### Statistical Analyses of Data

The data were analyzed by using Student’s unpaired *t*-test in the Microsoft Excel (ver.12.2.8). Differences were considered to be significant at *P* < 0.05 or *P* < 0.01.

## Supplementary information

**Additional file 1: Table S1.** Genome-edited rice lines (T_1_ generation). **Table S2.** Primers used in this study. **Fig. S1** Comparison of rice grain weight and protein concentration from wild-type Nipponbare and genome-edited lines (#8_1 and #8_8). Values represent means ± standard deviation from four independent rice samples. * and ** indicate *P* < 0.05 versus Ni and *P* < 0.01 versus Ni and #8_1, respectively. **Fig. S2** Semi-quantitative RT-PCR analysis of RNA extracted from seeds. PCR conditions were as follows: cycles of 95 °C 30 s, 60 °C 30 s, 72 °C 30 s were repeated by 27 times for *GAD3* and by 25 times for *TBP2*. **a***GAD3* as a target RNA; *TBP2*: TATA-binding Protein 2 as an internal control. **b** Relative expression of each *GAD3* determined by normalization of that of *TBP2*. **Fig. S3** Semi-quantitative RT-PCR analysis of RNA extracted from various tissues. PCR conditions were as follows: cycles of 95 °C 30 s, 60 °C 30 s, 72 °C 20 s were repeated by 24. **a***GAD1* (AB056060)*, GAD2* (AB056061), *GAD4* (AK101171), *GAD5* (AK070858) as a target RNA; *TBP2*: TATA-binding Protein 2 as an internal control. **b** Relative expression of each *GAD* determined by normalization of that of *TBP2*. **Fig. S4** Promoter activity of *OsGAD3* in rice. β-glucuronidase (GUS) reporter assays were performed in transgenic rice plants by introducing a promoter region of the *OsGAD3* gene (2868 bp from the initiation codon):: *GUS* gene, showing *GUS* expression at 1 week after germination of seedlings **a** to **c** and in seed **d**. **a**: lateral root, **b**: surface of leaf sheath, **c**: cross section of leaf sheath, **d**: transverse section of brown rice grain. Scale bars = 1 mm.

## Data Availability

The vectors used in this study will be available upon request.

## Abbreviations

bp: base pairs
C/N: Carbon/nitrogen
CaM: Calmodulin
CaMBD: Calmodulin-binding domain
CRISPR/Cas: Clustered regularly interspaced palindromic repeats/CRISPR-associated protein
GABA: γ-aminobutyric acid
GAD: Glutamate decarboxylase
GAPDH: Glyceraldehyde-3-phosphate dehydrogenase
gRNA: guide RNA
GUS: β-glucuronidase
Ni: Nipponbare
PAM: Protospacer adjacent motif
RT-PCR: Reverse transcriptase-polymerase chain reaction
TBP2: TATA-binding protein 2
UTR: Untranslated region
