# Supplementary material for: An In Vivo Targeted Deletion of the Calmodulin-Binding Domain from Rice Glutamate Decarboxylase 3 (OsGAD3) Increases γ-Aminobutyric Acid Content in Grains
Source: Rice (N Y). 2020 Mar 16;13:20. doi: 10.1186/s12284-020-00380-w (PMC7076103; doi:10.1186/s12284-020-00380-w)
Supplement: Supplementary file 1 — Additional file 1: Table S1. Genome-edited rice lines (T1 generation). Table S2. Primers used in this study. Fig. S1 Comparison of rice grain weight and protein concentration from wild-type Nipponbare and genome-edited lines (#8_1 and #8_8). Values represent means ± standard deviation from four independent rice samples. * and ** indicate P < 0.05 versus Ni and P < 0.01 versus Ni and #8_1, respectively. Fig. S2 Semi-quantitative RT-PCR analysis of RNA extracted from seeds. PCR conditions were as follows: cycles of 95 °C 30 s, 60 °C 30 s, 72 °C 30 s were repeated by 27 times for GAD3 and by 25 times for TBP2. aGAD3 as a target RNA; TBP2: TATA-binding Protein 2 as an internal control. b Relative expression of each GAD3 determined by normalization of that of TBP2. Fig. S3 Semi-quantitative RT-PCR analysis of RNA extracted from various tissues. PCR conditions were as follows: cycles of 95 °C 30 s, 60 °C 30 s, 72 °C 20 s were repeated by 24. aGAD1 (AB056060), GAD2 (AB056061), GAD4 (AK101171), GAD5 (AK070858) as a target RNA; TBP2: TATA-binding Protein 2 as an internal control. b Relative expression of each GAD determined by normalization of that of TBP2. Fig. S4 Promoter activity of OsGAD3 in rice. β-glucuronidase (GUS) reporter assays were performed in transgenic rice plants by introducing a promoter region of the OsGAD3 gene (2868 bp from the initiation codon):: GUS gene, showing GUS expression at 1 week after germination of seedlings a to c and in seed d. a: lateral root, b: surface of leaf sheath, c: cross section of leaf sheath, d: transverse section of brown rice grain. Scale bars = 1 mm. [file 12284_2020_380_MOESM1_ESM.pptx]

## Slide 1
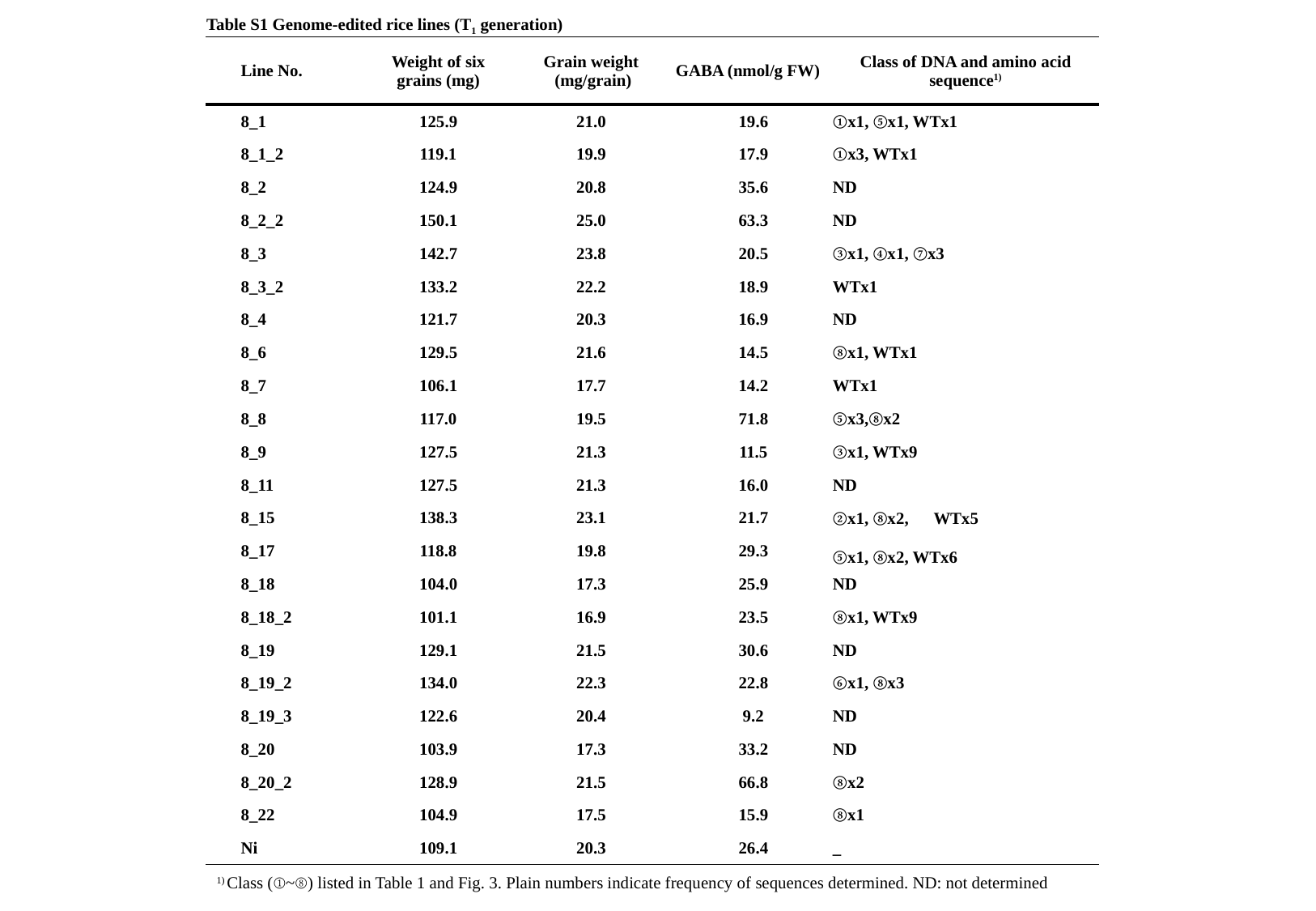

| Table S1 Genome-edited rice lines (T1 generation) | | | | | |
| --- | --- | --- | --- | --- | --- |
| | Line No. | Weight of six grains (mg) | Grain weight (mg/grain) | GABA (nmol/g FW) | Class of DNA and amino acid sequence1) |
| | 8\_1 | 125.9 | 21.0 | 19.6 | ①x1, ⑤x1, WTx1 |
| | 8\_1\_2 | 119.1 | 19.9 | 17.9 | ①x3, WTx1 |
| | 8\_2 | 124.9 | 20.8 | 35.6 | ND |
| | 8\_2\_2 | 150.1 | 25.0 | 63.3 | ND |
| | 8\_3 | 142.7 | 23.8 | 20.5 | ③x1, ④x1, ⑦x3 |
| | 8\_3\_2 | 133.2 | 22.2 | 18.9 | WTx1 |
| | 8\_4 | 121.7 | 20.3 | 16.9 | ND |
| | 8\_6 | 129.5 | 21.6 | 14.5 | ⑧x1, WTx1 |
| | 8\_7 | 106.1 | 17.7 | 14.2 | WTx1 |
| | 8\_8 | 117.0 | 19.5 | 71.8 | ⑤x3,⑧x2 |
| | 8\_9 | 127.5 | 21.3 | 11.5 | ③x1, WTx9 |
| | 8\_11 | 127.5 | 21.3 | 16.0 | ND |
| | 8\_15 | 138.3 | 23.1 | 21.7 | ②x1, ⑧x2,　WTx5 |
| | 8\_17 | 118.8 | 19.8 | 29.3 | ⑤x1, ⑧x2, WTx6 |
| | 8\_18 | 104.0 | 17.3 | 25.9 | ND |
| | 8\_18\_2 | 101.1 | 16.9 | 23.5 | ⑧x1, WTx9 |
| | 8\_19 | 129.1 | 21.5 | 30.6 | ND |
| | 8\_19\_2 | 134.0 | 22.3 | 22.8 | ⑥x1, ⑧x3 |
| | 8\_19\_3 | 122.6 | 20.4 | 9.2 | ND |
| | 8\_20 | 103.9 | 17.3 | 33.2 | ND |
| | 8\_20\_2 | 128.9 | 21.5 | 66.8 | ⑧x2 |
| | 8\_22 | 104.9 | 17.5 | 15.9 | ⑧x1 |
| | Ni | 109.1 | 20.3 | 26.4 | \_ |
1) Class (①~⑧) listed in Table 1 and Fig. 3. Plain numbers indicate frequency of sequences determined. ND: not determined

## Slide 2
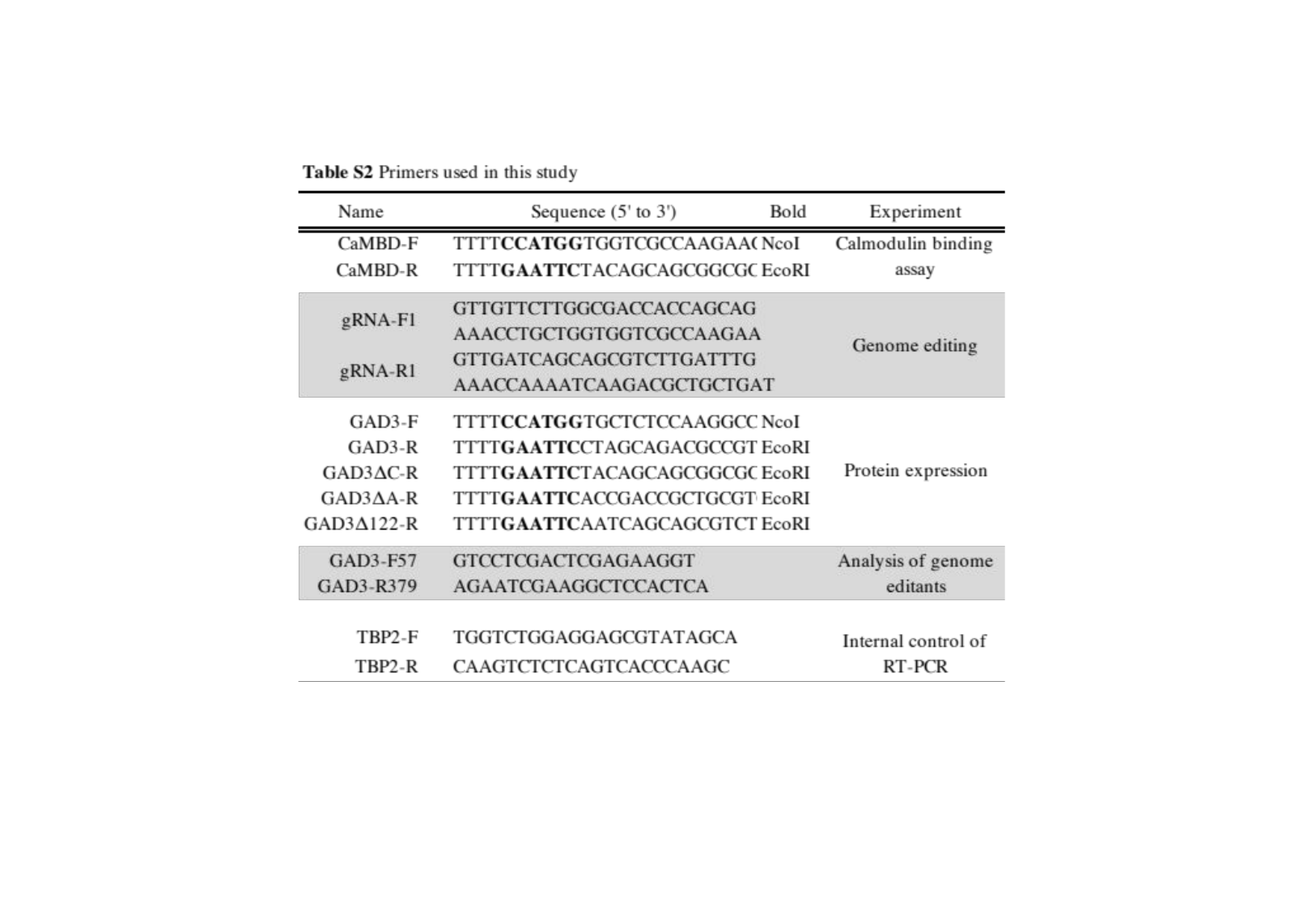

## Slide 3
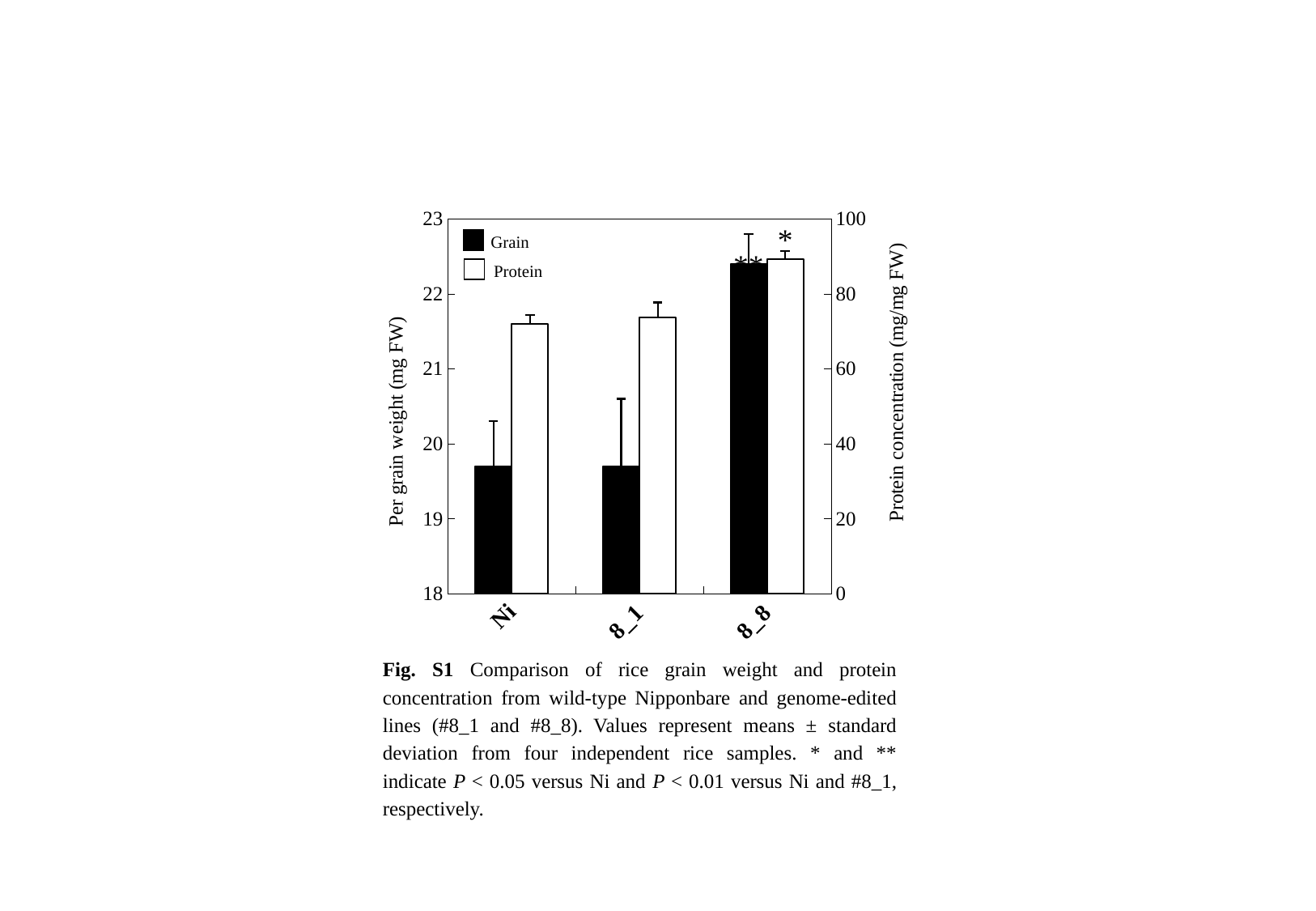

### Chart
| Category | 玄米重量 (mg) | | | タンパク質濃度 (ug/mg) |
|---|---|---|---|---|
| Ni | 19.7 | None | None | 72.0 |
| 8_1 | 19.7 | None | None | 73.8 |
| 8_8 | 22.4 | None | None | 89.3 |*
Grain
**
Protein
Fig. S1 Comparison of rice grain weight and protein concentration from wild-type Nipponbare and genome-edited lines (#8_1 and #8_8). Values represent means ± standard deviation from four independent rice samples. * and ** indicate P < 0.05 versus Ni and P < 0.01 versus Ni and #8_1, respectively.

## Slide 4
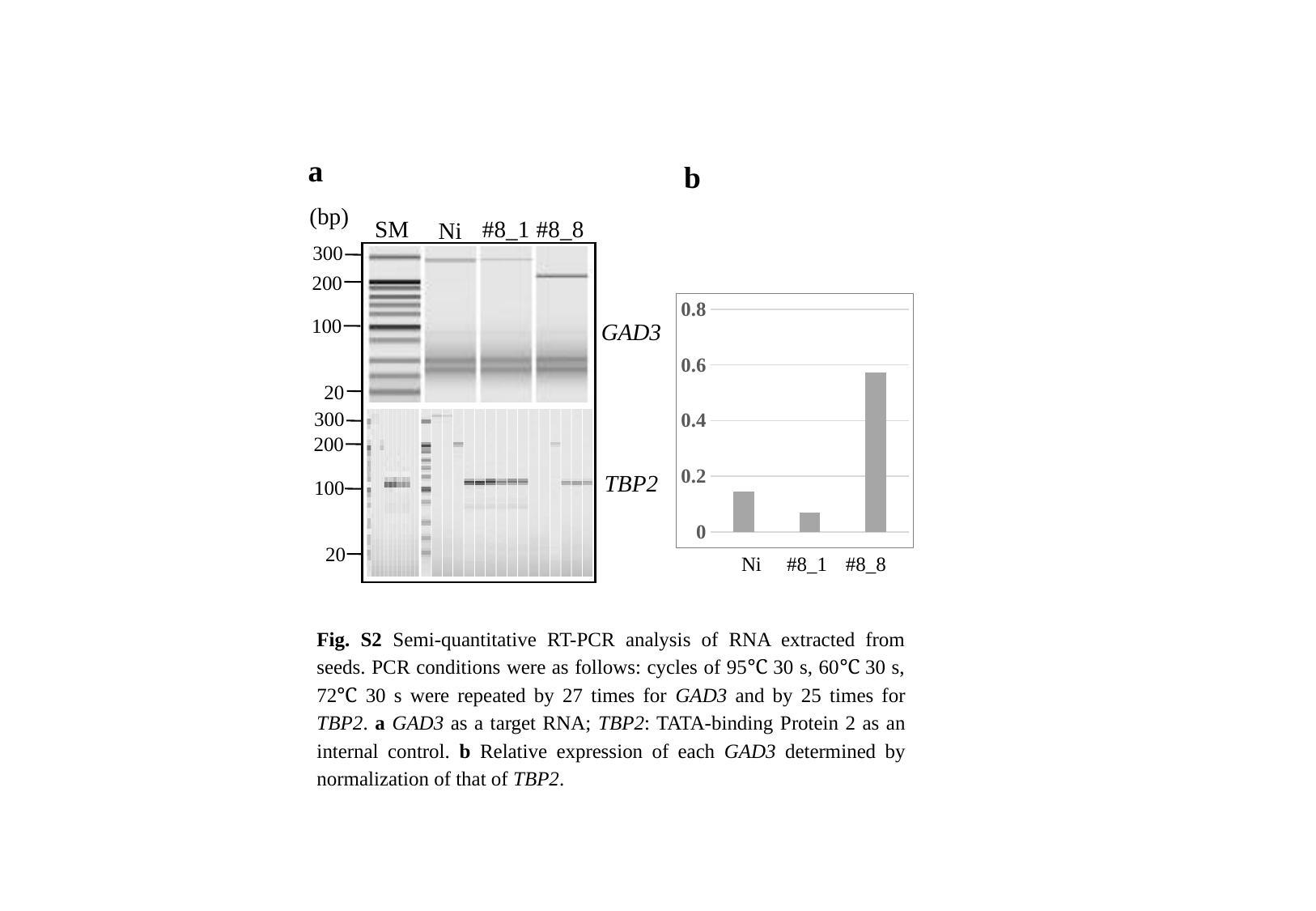

300
a
b
(bp)
#8_1
SM
#8_8
Ni
200
300
### Chart
| Category | |
|---|---|100
GAD3
20
200
TBP2
100
20
Ni
#8_1
#8_8
Fig. S2 Semi-quantitative RT-PCR analysis of RNA extracted from seeds. PCR conditions were as follows: cycles of 95℃ 30 s, 60℃ 30 s, 72℃ 30 s were repeated by 27 times for GAD3 and by 25 times for TBP2. a GAD3 as a target RNA; TBP2: TATA-binding Protein 2 as an internal control. b Relative expression of each GAD3 determined by normalization of that of TBP2.

## Slide 5
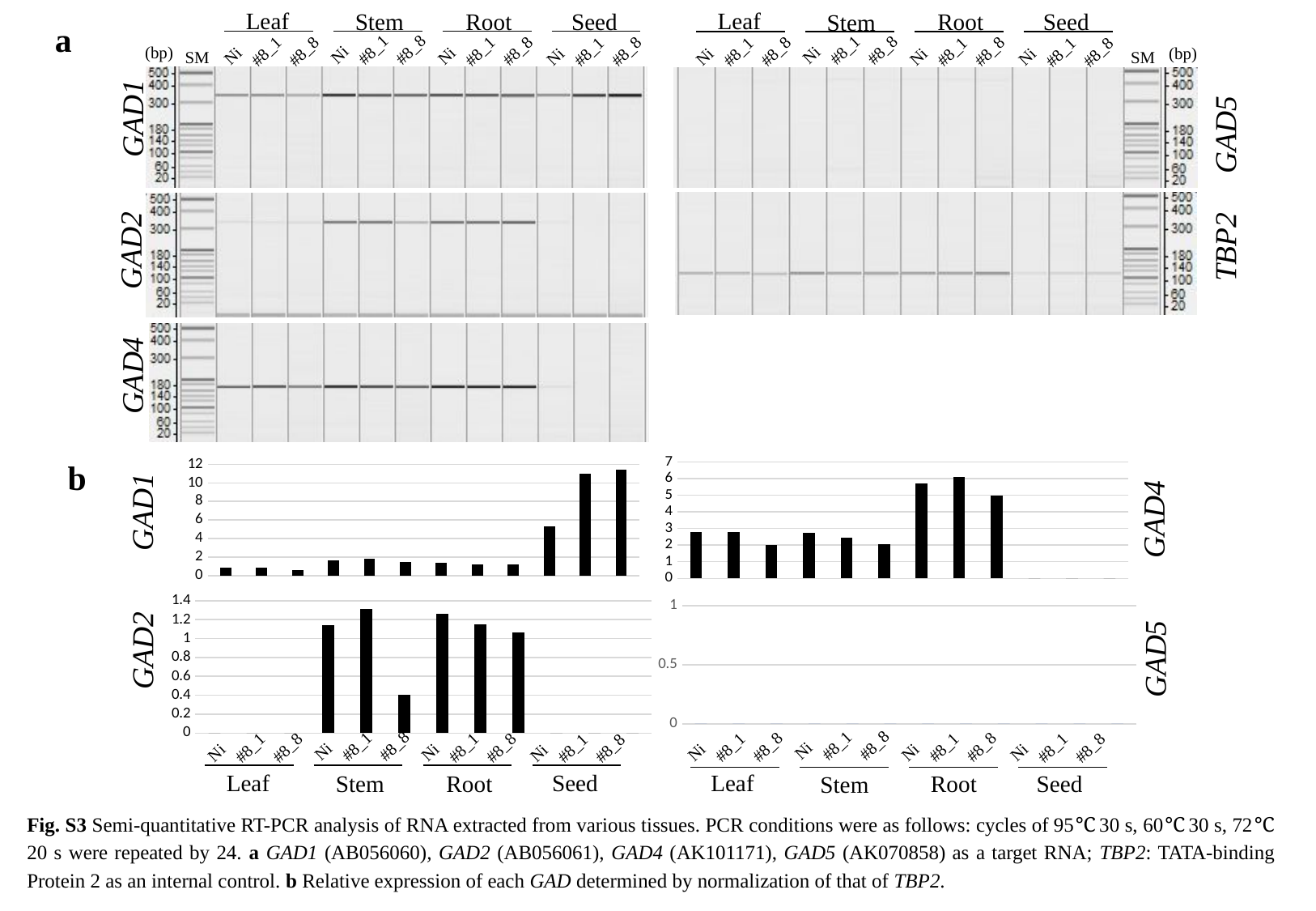

Leaf
Seed
Root
Stem
Fig. 4
Leaf
Seed
Root
Stem
#8_8
#8_1
#8_8
#8_8
#8_1
#8_1
#8_8
#8_1
Ni
Ni
Ni
Ni
a
#8_8
#8_1
#8_8
#8_8
#8_1
#8_1
#8_8
#8_1
Ni
Ni
Ni
Ni
(bp)
(bp)
SM
SM
GAD1
GAD5
TBP2
GAD2
GAD4
### Chart
| Category | |
|---|---|b
### Chart
| Category | |
|---|---|GAD1
GAD4
### Chart
| Category | |
|---|---|
### Chart
| Category | |
|---|---|GAD2
GAD5
#8_8
#8_1
#8_8
#8_8
#8_1
#8_1
#8_8
#8_1
Ni
Ni
Ni
Ni
#8_8
#8_1
#8_8
#8_8
#8_1
#8_1
#8_8
#8_1
Ni
Ni
Ni
Ni
Leaf
Seed
Root
Stem
Leaf
Seed
Root
Stem
Fig. S3 Semi-quantitative RT-PCR analysis of RNA extracted from various tissues. PCR conditions were as follows: cycles of 95℃ 30 s, 60℃ 30 s, 72℃ 20 s were repeated by 24. a GAD1 (AB056060), GAD2 (AB056061), GAD4 (AK101171), GAD5 (AK070858) as a target RNA; TBP2: TATA-binding Protein 2 as an internal control. b Relative expression of each GAD determined by normalization of that of TBP2.

## Slide 6
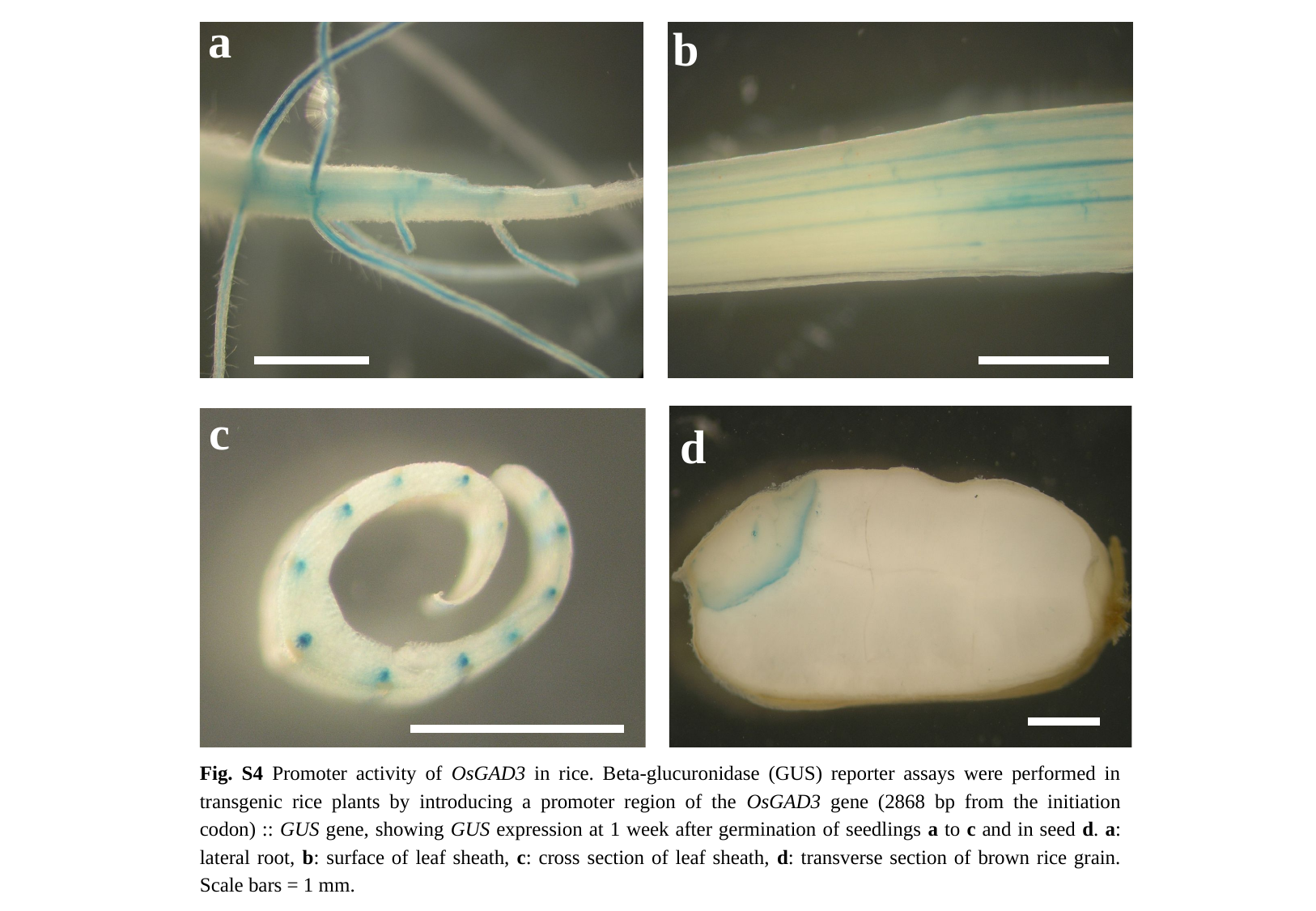

a
b
c
d
Fig. S4 Promoter activity of OsGAD3 in rice. Beta-glucuronidase (GUS) reporter assays were performed in transgenic rice plants by introducing a promoter region of the OsGAD3 gene (2868 bp from the initiation codon) :: GUS gene, showing GUS expression at 1 week after germination of seedlings a to c and in seed d. a: lateral root, b: surface of leaf sheath, c: cross section of leaf sheath, d: transverse section of brown rice grain. Scale bars = 1 mm.
